# Metallic Materials: Structure Transition, Processing, Characterization and Applications

**DOI:** 10.3390/ma17050985

**Published:** 2024-02-21

**Authors:** Jing Hu, Ze He, Xiliang Liu

**Affiliations:** 1 Jiangsu Key Laboratory of Materials Surface Science and Technology, National Experimental Demonstration Center for Materials Science and Engineering, Changzhou University, Changzhou 213164, China; heze@cczu.edu.cn (Z.H.); liuxiliang@cczu.edu.cn (X.L.); 2 Huaide College, Changzhou University, Jingjiang 214500, China

This Special Issue provides readers with up-to-date information on the recent progress in the structure transition, processing, characterization, and applications of metals, including ferrous and nonferrous metals. The contents include the following aspects in manufacturing processes and properties/performance: enhancing the properties of metals by advanced element design; novel heat treatment technology; novel surface modification technology; novel methodologies for characterization of the microstructure and properties; and novel processing technology.

This Special Issue aims to comprehensively present the latest research findings, methodologies, and crucial insights on the topic “Metallic Materials: Structure Transition, Processing, Characterization and Applications” from leading researchers and practitioners in this field. The contributions include original scientific work concerned with fundamental research and applied aspects of applications of metals.

Our contributors, comprising outstanding researchers, scholars, and industrial experts, have brought forth advanced perspectives to enhance our collective understanding of the structure transition, processing, characterization, and applications of metals. Studies of metals are gaining significant progress not only due to the findings of novel composition designs, but also due to performance enhancements by heat treatment and surface modification [1,2,3,4,5]. The unique properties of metals include their high strength, making them able to bear the needed loads, and their ductility, which makes them readily formable into practical shapes and permits small amounts of yielding to sudden and severe loads. Moreover, advanced heat treatment and surface modification broaden the application range of metals, such as at very low and ultra-high temperatures, in corrosive environments, etc., which not only makes a significant contribution to their durability and overall performance but also enhances the environmental sustainability of metals [6,7,8,9,10]. Research efforts also include predicting the properties of metals after different processes of casting, deforming, and heat treating by using machine learning and software [11,12,13,14,15]. Despite these advancements, there is an ongoing need for fundamental research, standardization, and specification improvements for metals [16,17,18,19,20].

There are twelve research papers published in this Special Issue, covering less than two years, and more than 100 authors from many institutions and industries contributed to the published papers. In order to express the high quality of the efforts and progress that these outstanding authors have made, all twelve research papers are briefly summarized below.

Ke et al. [21] contributed a paper entitled “Development of Y_2_O_3_ Dispersion-Strengthened Copper Alloy by Sol-Gel Method”, which demonstrates an oxide dispersion-strengthened Cu alloy with a content of 1 wt.% nanoscale and uniformly distributed Y_2_O_3_ particles in an ultra-fine-grained Cu matrix fabricated through citric acid sol–gel synthesis and spark plasma sintering (SPS). The unique microstructure provides excellent mechanical properties with a tensile strength of 572 MPa and a total elongation of 6.4%, together with high thermal conductivity.

Pugliese et al. [22] contributed a paper entitled “The Local Structure and Metal-Insulator Transition in a Ba_3_Nb_5−x_Ti_x_O_15_ System”, which illustrates a local structure of a filled tetragonal tungsten bronze (TTB) niobate Ba_3_Nb_5−x_Ti_x_O_15_ (x = 0, 0.1, 0.7, 1.0), showing a metal–insulator transition with Ti substitution, having a substantial effect on the local structure.

Xie et al. [23] contributed a paper entitled “Characterization of Carbide Precipitation during Tempering for Quenched Dievar Steel”, in which they found that the carbide precipitation sequence on tempering is M_8_C_7_ + cementite → M_8_C_7_ + M_2_C + M_7_C_3_ → M_8_C_7_ + M_7_C_3_ + M_23_C_6_ → M_8_C_7_ + M_7_C_3_ + M_23_C_6_ + M_6_C; the sizes for inter-lath carbides increased noticeably with increasing tempering temperature, whereas the sizes for intra-lath carbides remained nearly constant.

Hu et al. [24] contributed a paper entitled “Effect of Pulsed Magnetic Field on the Microstructure of QAl9-4 Aluminium Bronze and Its Mechanism”, in which it was found that the dislocation density, grain boundary angle, and microhardness of the alloy significantly decreased after the magnetic field treatment; this may have resulted from the transition to the electronic energy state at the site of dislocation pinning caused by the pulsed magnetic field, leading to free movement of the vacancy or impurity atoms.

Xu et al. [25] contributed a paper entitled “The Effect of Novel Complex Treatment of Annealing and Sandblasting on the Microstructure and Performance of Welded TA1 Titanium Plate”, in which it was found that the novel complex treatment had an efficient effect on regulating the microstructure of the weld zone and making the microstructure in the weld zone close to that of the base metal. An application test confirmed that the adverse impact of a longitudinal weld on the quality of electrolytic copper foil could be resolved by adopting this novel complex treatment.

Wu et al. [26] contributed a paper entitled “Microstructure and High-Temperature Ablation Behaviour of Hafnium-Doped Tungsten-Yttrium Alloys”, in which it was found that the properties of the microstructure and high-temperature ablation behavior of hafnium-doped tungsten–yttrium alloy can be improved evidently by adding an appropriate amount of hafnium. The alloy exhibited high stability and excellent ablation resistance with a hafnium content of 20 wt.%.

Zhang et al. [27] contributed a paper entitled “Towards an Optimized Artificial Neural Network for Predicting Flow Stress of In718 Alloys at High Temperatures”, in which it was found that an ANN with one hidden layer and four nodes possessed optimized performance for predicting the flow stress of In718 alloys.

Newishy et al. [28] contributed a paper entitled “Friction Stir Welding of Dissimilar Al 6061-T6 to AISI 316 Stainless Steel: Microstructure and Mechanical Properties”, in which the authors welded dissimilar butt joints between 6061-T6 aluminum alloy and AISI 316 stainless steel by FSW using different processing parameters. They found that significant continuous dynamic recrystallization (CDRX) occurred in the stir zone (SZ) of the Al side, while the steel underwent severe deformation and discontinuous dynamic recrystallization (DDRX).

Liu et al. [29] contributed a paper entitled “Revisiting the High-Pressure Behaviors of Zirconium: Nonhydrostaticity Promoting the Phase Transitions and Absence of the Isostructural Phase Transition in β-Zirconium”, which discovered that both the purity and the stress state of the sample (the grade of hydrostaticity/nonhydrostaticity) affect the PT pressure of Zr. The stress state is the dominant factor, and nonhydrostaticity significantly promotes the PT of Zr.

Chen et al. [30] contributed a paper entitled “Effect of the Solid Solution and Aging Treatment on the Mechanical Properties and Microstructure of a Novel Al-Mg-Si Alloy”, in which it was found that the best strengthening effect can be achieved when the solubility of the MgSi phase and precipitate â″(Mg2Si phase) is at its maximum. The aging strengthening of alloys is a comprehensive effect of the dislocation cutting mechanism and bypass mechanism, with the joint effect of diffusion strengthening of Al3(Er,Zr) particles and the addition of Er and Zr elements promoting the precipitation strengthening of â″ phases.

Chen et al. [31] contributed a paper entitled “Improved Analytical Model for Thermal Softening in Aluminum Alloys Form Room Temperature to Solidus”, which proposed an analytical model for describing the thermal softening of aluminum alloys from room temperature to solidus temperature, in which the concept of temperature-dependent transition between two thermal softening regimes was implemented. The proposed model compared favorably to the well-known Sellars–Tegart model and Johnson–Cook model.

Zhuang et al. [32] contributed a paper entitled “Mechanism Analysis for the Enhancement of Low-Temperature Impact Toughness of Nodular Cast Iron by Heat Treatment”, which explored the enhancement mechanism of the low-temperature impact toughness of nodular cast iron by heat treatment. It was found that heat treatment brought about a significant decrease in high-angle grain boundaries (HAGB) between 59° and 60°.

This Special Issue highlights the synergy across academia, research institutions, and industries, confirming their pivotal role in the continuous advancement of this field. We extend our sincere gratitude to all the authors who contributed to this Special Issue. Their valuable research contributions made this Special Issue possible, and we genuinely appreciate all the efforts they made.

In conclusion, we hope that this Special Issue serves as a comprehensive resource, fostering innovation and dialogue among researchers, engineers, and industry experts in this dynamic field.

For further information, readers are encouraged to refer to the complete articles.
